# The origin of eukaryotes and rise in complexity were synchronous with the rise in oxygen

**DOI:** 10.3389/fbinf.2023.1233281

**Published:** 2023-09-01

**Authors:** Jack M. Craig, Sudhir Kumar, S. Blair Hedges

**Affiliations:** ^1^ Center for Biodiversity, Temple University, Philadelphia, PA, United States; ^2^ Institute for Genomics and Evolutionary Medicine, Temple University, Philadelphia, PA, United States; ^3^ Department of Biology, Temple University, Philadelphia, PA, United States

**Keywords:** eukaryotes, phylogeny, dating, biological complexity, tree of life, great oxidation event, molecular clock, prokaryotes

## Abstract

The origin of eukaryotes was among the most important events in the history of life, spawning a new evolutionary lineage that led to all complex multicellular organisms. However, the timing of this event, crucial for understanding its environmental context, has been difficult to establish. The fossil and biomarker records are sparse and molecular clocks have thus far not reached a consensus, with dates spanning 2.1–0.91 billion years ago (Ga) for critical nodes. Notably, molecular time estimates for the last common ancestor of eukaryotes are typically hundreds of millions of years younger than the Great Oxidation Event (GOE, 2.43–2.22 Ga), leading researchers to question the presumptive link between eukaryotes and oxygen. We obtained a new time estimate for the origin of eukaryotes using genetic data of both archaeal and bacterial origin, the latter rarely used in past studies. We also avoided potential calibration biases that may have affected earlier studies. We obtained a conservative interval of 2.2–1.5 Ga, with an even narrower core interval of 2.0–1.8 Ga, for the origin of eukaryotes, a period closely aligned with the rise in oxygen. We further reconstructed the history of biological complexity across the tree of life using three universal measures: cell types, genes, and genome size. We found that the rise in complexity was temporally consistent with and followed a pattern similar to the rise in oxygen. This suggests a causal relationship stemming from the increased energy needs of complex life fulfilled by oxygen.

## Introduction

Life arose early in Earth’s history (∼4 Ga), quickly giving rise to prokaryotes (Bacteria and Archaea) (Knoll and Nowak, 2017). Eukaryotes, synonymous with “complex life,” arose later and are characterized by distinct organelles and the capacity for multicellularity with diverse cell types. Molecular clocks have yielded a wide range of times for the origin of eukaryotes, from 2.10 Ga (Shih and Matzke, 2013) to 0.91 Ga (Betts et al., 2018), and the earliest widely accepted fossils are dated to 1.62 Ga (Knoll and Nowak, 2017). The evolution of complex eukaryotic life has frequently been associated with the Great Oxidation Event (GOE), 2.43–2.22 Ga (Poulton et al., 2021), when global atmospheric oxygen reached a sustained presence above 10^−5^ times its present level. This is because oxygen would have provided a rich new energy source for the first eukaryotes, having acquired mitochondria capable of aerobic respiration (Sagan, 1967), and thus served as a catalyst for complex life (Bonner, 2009; Lane and Martin, 2010; Schavemaker and Muñoz-Gómez, 2022). However, if the youngest molecular clock estimates for the origin of eukaryotes are to be believed, then the GOE would have predated eukaryogenesis by as much as one billion years. This apparent temporal decoupling has been the major reason why some have concluded that the origin and initial diversification of eukaryotes was unrelated to oxygen (Betts et al., 2018; Mills et al., 2022). Here, we focus on the timing of eukaryogenesis to determine whether or not it is temporally decoupled from the rise in atmospheric oxygen.

**FIGURE 1:**
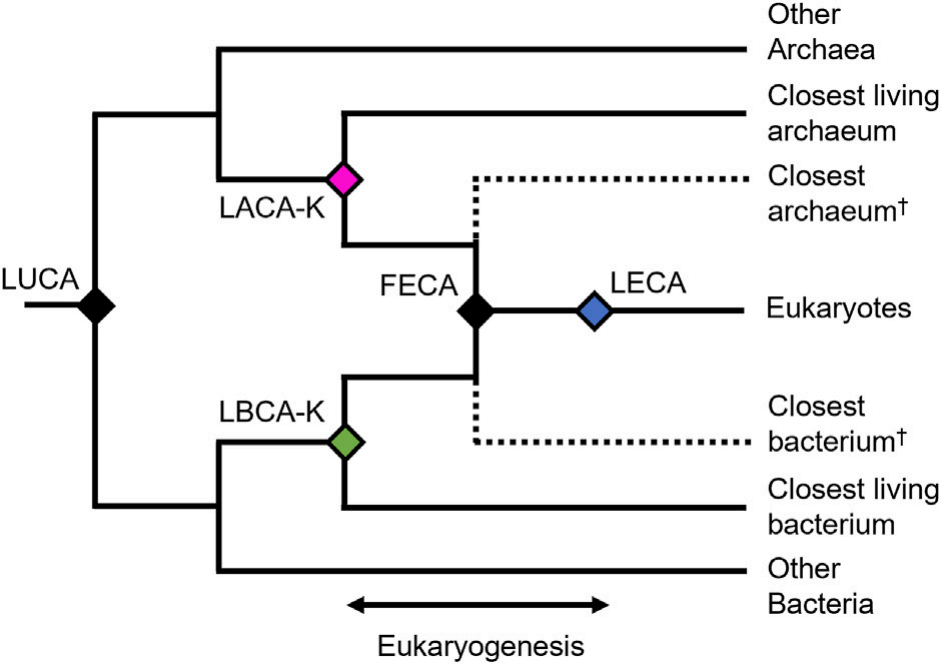
Phylogenetic hypothesis for the origin of eukaryotes. The last universal common ancestor (LUCA) diverged into Archaea and Bacteria. Subsequently the first eukaryotic common ancestor (FECA) formed by the endosymbiosis of the last archaeal and last bacterial common ancestors of eukaryotes (LACA-K and LBCA-K, respectively.

In order to establish a reliable timeframe for eukaryogenesis, it is important to consider the appropriate set of evolutionary events. From a phylogenetic standpoint (Figure 1), we interpret eukaryogenesis to have occurred at the stem eukaryote node, which captures the formation of the first eukaryotic common ancestor (FECA) from an endosymbiosis in which a bacterial symbiont gave rise to an organelle within an archaeal host (Archibald, 2015; Lazcano and Peretó, 2021). Importantly, the resulting organism had a hybrid genome similar to that of most living eukaryotes, with large numbers of genes from the host and symbiont. While there may have been a series of gene transfer events leading to the formation of modern eukaryotes, each conferring traits now associated with modern eukaryotic life (Strassert et al., 2021), we interpret the most recent of these as having given rise to FECA. Furthermore, theoretical eukaryotic life prior to FECA, lacking the defining characteristics of modern eukaryotes such as the mitochondrion, may be difficult enough to distinguish from contemporary prokaryotic life as to render FECA the first unequivocally-eukaryotic organism.

However, we cannot directly time eukaryogenesis as we would a typical phylogenetic divergence event. FECA is not tied to a single phylogenetic divergence, but rather one or more fusions, and so its age cannot be inferred phylogenetically, at least directly. Furthermore, we are limited by the incompleteness of our current phylogenetic knowledge, as the two ancient species which underwent endosymbiosis are likely now extinct. In their absence, we must determine the shortest interval within which the process of eukaryogenesis occurred using living taxa.

To establish the older boundary of this interval, we assume that if eukaryogenesis is the result of an archaeal host accepting a bacterial endosymbiont, then the divergence of both the archaeal and bacterial organisms from their closest relatives necessarily preceded the fusion event which resulted in the first eukaryote (FECA). This is most simply understood if we accept a single fusion event resulting in the eukaryotic mitochondrion arising from a bacterial endosymbiont, but any theory of serial endosymbiosis with multiple transfer events still necessarily places the divergences of each organism from their nearest relatives as a chronological prerequisite for any genetic transfer. Therefore, we establish the older boundary of our eukaryogenesis interval as the divergence time between eukaryotes and either their last bacterial common ancestor (LBCA-K) or their last archaeal common ancestor (LACA-K), whichever is younger (Figure 1). Since both divergences must have happened prior to eukaryogenesis, choosing the younger of the two allows us more precision in constraining the interval.

The younger boundary of the eukaryogenesis interval may be defined simply as the earliest evidence of eukaryotes, whether as a widely accepted eukaryotic fossil, or as the phylogenetic crown node of the eukaryotes, which is inferred to be the last eukaryotic common ancestor (LECA). While LECA is defined phylogenetically as the youngest organism to which all living eukaryotes may trace their lineage, it is likely that time passed between the formation of FECA by endosymbiosis and the later evolution of LECA. During this time, many organisms which would be recognizable as eukaryotes may have evolved and subsequently gone extinct, but left no evidence in the fossil record. Thus, we can only be sure that eukaryogenesis occurred no later than LECA, and therefore the true age of FECA is, at a minimum, older than or equal to that of LECA.

So while it remains impossible to precisely time the emergence of the first eukaryote, we can be confident it happened in between the divergence of its archaeal and bacterial ancestors from their relatives, and the time of the oldest conclusive evidence of its existence, either from phylogenetic or fossil evidence. We further cannot be certain of the process of eukaryogenesis, be it a singular or serial endosymbiosis, or of the precise nature of the two participants, but by establishing the time interval as we have here, we capture the evolution of FECA agnostic to the active debate on this subject.

To test whether the origin and diversification of eukaryotes was coupled with the rise in oxygen, we use genes of archaeal origin, typical in studies of molecular dating, and genes of bacterial origin, which are rarely used. Genes of bacterial origin are advantageous because they provide access to a better (closer in time) maximum constraint on eukaryogenesis, the LBCA-K node (Figure 1). We also reconstruct the rise in complexity using three universal metrics. We do this to determine how closely the rise in complexity matches the rise in oxygen. In both the timing of eukaryogenesis and the rise in complexity, we do not find evidence of uncoupling. Instead, the results support a synchronicity between oxygen and the evolution of eukaryotes.

### Timing eukaryogenesis

**FIGURE 2:**
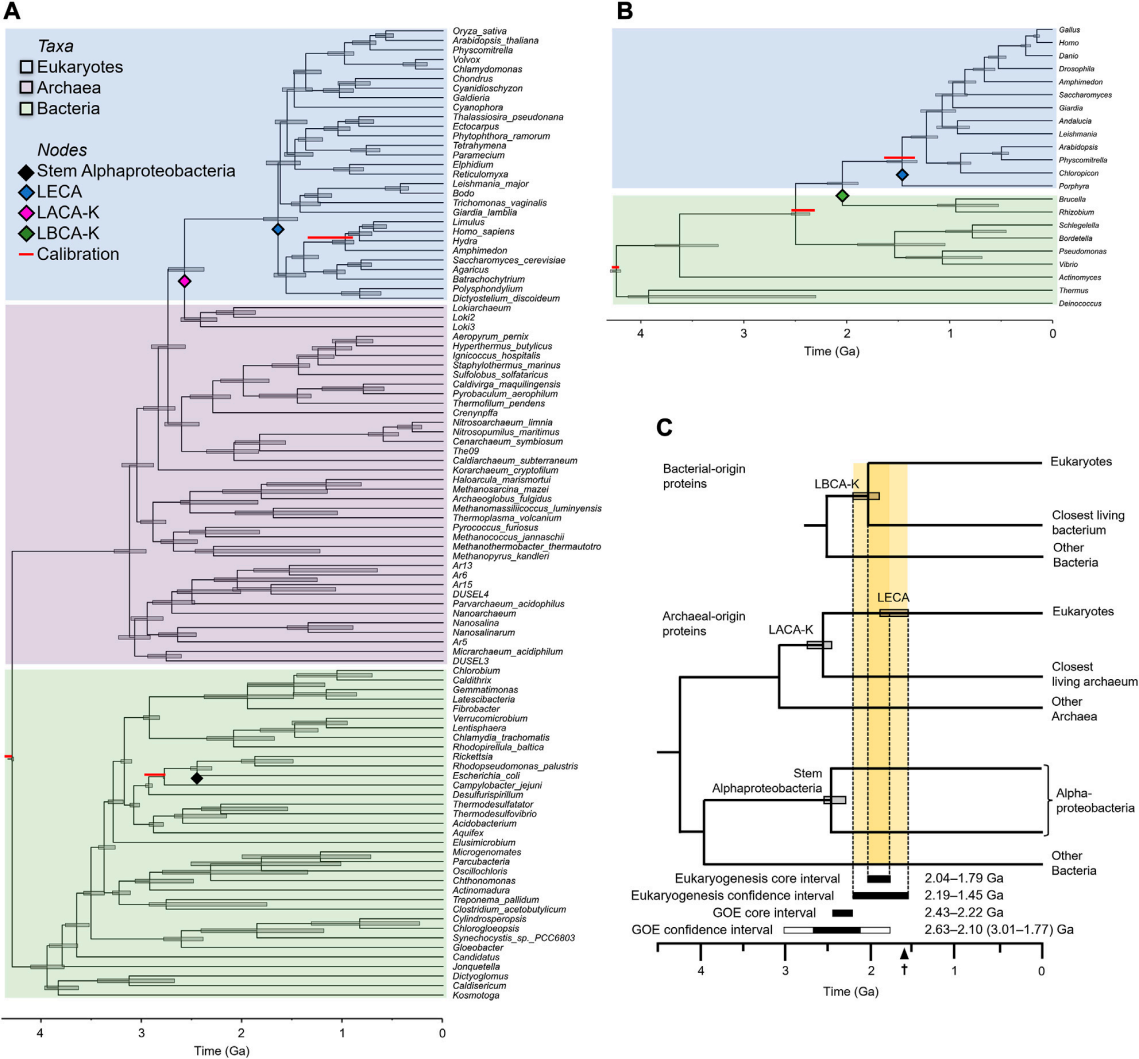
Inferring the eukaryogenesis interval. **(A)** Phylogeny derived from eukaryotic proteins of archaeal origin. **(B)** Phylogeny derived from eukaryotic proteins of bacterial origin. Red bars above nodes indicate literature consensus calibrations. Note that the divergence most proximate to eukaryogenesis, between either LACA-K or LBCA-K and eukaryotes, is never calibrated. For the phylogeny of bacterial proteins, the minimal calibration scheme is not shown, but resulted in nearly identical estimates of the eukaryogenesis interval (see Methods). **(C)** Summary timetree showing the inferred time intervals for eukaryogenesis and the Great Oxidation Event (GOE). The core eukaryogenesis interval is bounded by the major constraining mean times for LBCA-K and LECA whereas the conservative eukaryogenesis interval also considers the 95% confidence intervals on those dates. The core GOE interval is bounded by the consensus of environmental proxy dates (Poulton et al., 2021) whereas the conservative GOE interval also considers the 95% confidence intervals (CIs) on those dates (Hodgskiss and Sperling, 2022), with bounds determined by (left to right) Nonzero I/(Ca + Mg) (3.01 Ga), red beds (2.63 Ga), MIF-S (2.10 Ga), and RSDM (1.77 Ga); all using the optimal linear estimation method. The earliest eukaryotic fossil (1.62 Ga) is indicated on the scale bar.

Proteins of bacterial-origin are assumed to have been transferred to an archaeal host during eukaryogenesis, making them promising candidates for timing this event. But previous efforts to resolve the early history of eukaryotes using these genes have been inconclusive. This is due to difficulty identifying homologous proteins and accounting for their rapid rate of evolution which obscures the true phylogenetic signal over two billion years of evolution (Hedges et al., 2001; Derelle and Lang, 2012; He et al., 2014). As a result, the sequence of branching among basal eukaryotic clades remains actively debated (Chernikova et al., 2011; Derelle and Lang, 2012; He et al., 2014; Vidaurri, 2020). Therefore, to avoid any taxonomic confusion, which would further complicate our dating effort, we constructed a timetree of a minimal taxon set including 22 well-studied species proximate to eukaryogenesis based on 31 eukaryotic proteins of bacterial origin. These proteins were inferred to be bacterial in origin because they were primarily located in the mitochondrion and their function was associated with the production of cellular energy, following a consensus (Roger et al., 2017; Martijn et al., 2018). We timed the interval with a set of consensus calibrations derived from TimeTree (Kumar et al., 2022) (Table 1, Supplementary Tables S1, S2), which has shown promise in calibrating difficult nodes where fossils are not available (Powell et al., 2020). Because this tree was constructed exclusively from proteins contributed by the bacterial symbiont to the first eukaryote, it supports Alphaproteobacteria as the nearest relative of eukaryotes (the LBCA-K), in contrast to more common phylogenies of genes derived from the archaeal host, characterized by an archaeal closest relative. Based on this tree, we estimated the divergence between eukaryotes and LBCA-K to have occurred 2.04 (2.19–1.89) Ga (Figure 2).

**TABLE 1:** Calibration scheme used for the phylogeny of proteins of bacterial origin. Both minimal and complete schemes are shown. Topology follows literature consensus.

Calibration (topology follows literature consensus)	Older boundary (bya)	Younger boundary (bya)	Number of studies	Included in minimal calibration scheme?
Last Universal Common Ancestor (LUCA)	4.30	4.19	3	Yes
Terrabacteria (outgroup)	2.83	2.48	2	No
Proteobacteria + Eukaryota (ingroup)	2.54	2.33	4	Yes
Betaproteobacteria + Gammaproteobacteria	2.12	1.66	3	No
Eukaryota crown	1.64	1.30	7	Yes
Opisthokonta crown	1.41	1.05	15	No
Metazoa crown	1.31	0.87	10	No
Alpaproteobacteria crown	0.60	0.58	2	No
Amniota	0.32	0.32	28	No

We also constructed a timetree of 102 species based on 28 eukaryotic proteins inferred to have originated in the archaeal ancestor of eukaryotes. Proteins of archaeal origin, the most conserved of which are treated as “core” genes (Charlebois and Doolittle, 2004; Bapteste et al., 2008), are often functionally associated with the maintenance and transcription of DNA. Inferences of the phylogeny of these genes will necessarily recover eukaryotes nested within the Archaea. Rather than assemble this alignment *de novo*, we took a published data-rich alignment (Betts et al., 2018), filtered it to only the most conserved sites to account for the ancient time scale (Castresana, 2000), and applied a set of consensus calibrations (Table 2, Supplementary Table S2). Based on this tree, we estimated the stem divergence among the Asgard group of Archaea (the LACA-K) to have occurred 2.58 (2.74–2.38) Ga and LECA to have diverged 1.65 (1.79–1.45) Ga (Figure 2). Given that LACA-K formed the host in the endosymbiotic event leading to modern eukaryotes, this earlier date than the interval derived from proteins of bacterial origin is expected.

**TABLE 2:** Calibration scheme used for the phylogeny of proteins of archaeal origin. Topology follows literature consensus.

Calibration (topology follows literature consensus)	Older boundary (bya)	Younger boundary (bya)	Number of studies
Last Universal Common Ancestor (LUCA)	4.30	4.19	3
Proteobacteria + *Campylobacter* (older)	2.97	2.78	2
Metazoa crown (younger)	1.31	0.87	10

From these two sets of dates, we establish a conservative eukaryogenesis interval defined by the youngest possible well-constrained older boundary and the oldest possible well-constrained younger boundary (Figure 2C). To avoid false precision, we take the older 95% HPD (highest posterior density) of our LBCA-K estimate as our older boundary, and the youngest boundary of the archaeal-gene estimate of LECA as our younger boundary, resulting in a conservative eukaryogenesis interval of 2.19–1.45 Ga. This 0.64 billion-year molecular clock interval, although large, is 54% shorter than the 1.19 billion-year interval (2.10–0.91 Ga) derived in two previous studies (Shih and Matzke, 2013; Betts et al., 2018).

Although the conservative eukaryogenesis interval is important, it does not convey the distribution of probability within that interval. For this, we establish a “core” eukaryogenesis interval defined by the maximum bound, LBCA-K (2.04 Ga), and the minimum bound, LECA (1.79 Ga), estimated from our phylogeny of archaeal-origin proteins. This narrow core interval (2.04–1.79 Ga) represents the most probable time of eukaryogenesis within the broader conservative interval of 2.19–1.45 Ga.

The fossil record can be used to test these two intervals. Independently verified, uncontested eukaryotic fossils dated to at least 1.62 Ga (Lamb et al., 2009; Li et al., 2013; Knoll and Nowak, 2017) are slightly older than the minimum bound of our conservative eukaryogenesis interval, 1.45 Ga. and slightly younger than that of our narrower core interval, at 1.79 Ga.

This fossil suggests that our conservative interval may be slightly too broad, as it provides evidence of the evolution of eukaryotes 0.17 Ga earlier than the younger boundary, but it also falls 0.17 Ga outside of our core interval. Thus, the existence of fossilized eukaryotic life dated to 1.62 Ga supports our core eukaryogenesis interval, while suggesting that our broader, more conservative interval may be slightly too cautious (Figure 2C).

### Biological complexity

The ability of eukaryotic life to derive energy from atmospheric oxygen has been proposed in earlier work as a catalyst for their rise in complexity (Sagan, 1967; Hedges et al., 2004; Bonner, 2009). Based on this premise, we expect to see a rise in global complexity contemporary with the GOE and eukaryogenesis. However, while complexity has been rigorously investigated within some clades of eukaryotes using traits such as body size, these metrics are not compatible with all forms of life, limiting the scope of these analyses. Complexity has only been examined previously across the full tree of life using cell types as a singular metric (Hedges et al., 2004).

**FIGURE 3:**
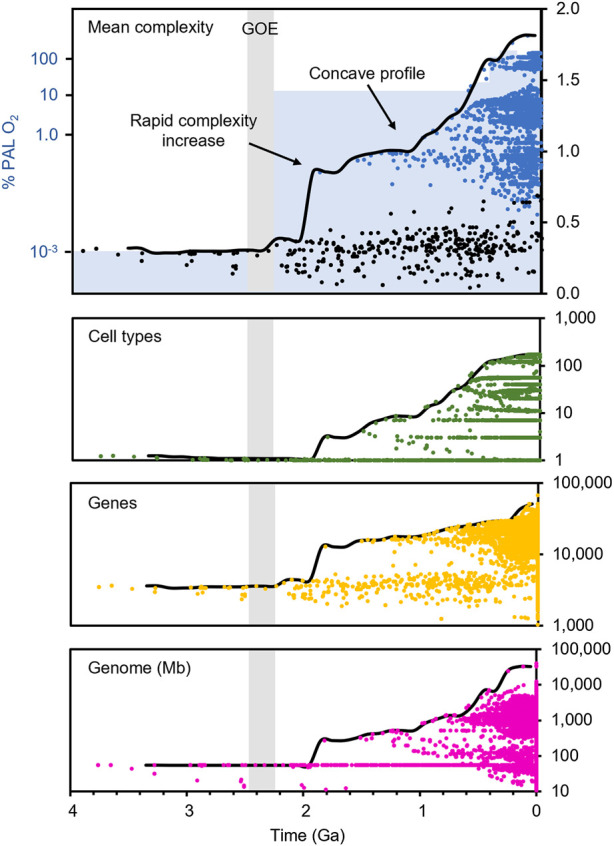
Complexity through time across the tree of life. Reconstructed mean complexity (top panel, black = prokaryotes, blue = eukaryotes), the number of unique cell types (second panel, green), the number of genes (third panel, gold), and the size of the genome in megabases (fourth panel, magenta). Complexity of extant organisms (tips, time zero) not shown for the top panel as it obscures the axis. Timescale from TimeTree (Kumar et al., 2022). Pale gray bar indicates the GOE core interval (2.43–2.22 Ga). Blue shading indicates maximum most likely atmospheric oxygen concentration relative to current levels (% PAL O_2_).

We analyzed complexity data across the tree of life by first obtaining a supertree built from over 4,000 published phylogenies including over 137,000 species (Kumar et al., 2022). We reconstructed the ancestral states of three universal measures of complexity at the family level: the number of unique cell types, the total number of genes, and the size of the genome in megabases, to generate a single metric of mean complexity through time (Figure 3).

All three individual measures of complexity, as well as the combined metric, show a rapid increase at LECA, the earliest node among living eukaryotes. While some single-measure estimates are inherently biased by the nature of reconstruction across a vast, incompletely-sampled phylogeny, the overall trend is unmistakable: the first eukaryotic organisms are substantially more complex than their prokaryotic contemporaries, and the increasing trend in global maximum complexity is driven by eukaryotes for the next two billion years (Figure 3). The shape of the complexity profile, with its rapid early rise and later (Neoproterozoic and Phanerozoic) additional rise, creating a concave profile, is consistent with the two-step rise in oxygen (Figure 3).

## Discussion

Our estimated eukaryogenesis interval falls within 200 million years of the GOE, calling into question recent claims (Betts et al., 2018; Mills et al., 2022) that the two events are temporally uncoupled. While the time between the end of the GOE and the onset of our eukaryogenesis interval is measurable in hundreds of millions of years, it is only 7% of the total time since the end of the GOE. Therefore, our results suggest that complex eukaryotic life has been diversifying on Earth for more than 90% of the time during which global atmospheric oxygen has been present in significant levels (Figure 3).

We believe that there are at least four explanations for claims that oxygen did not play a role in eukaryogenesis: 1) the use of calibration methodology in multiple molecular clock analyses that biased the results towards the recent, 2) the use of the last common ancestor of living eukaryotes (LECA) as a proxy for the origin of eukaryotes, 3) the lack of consideration of environments (e.g., shallow water rift zones), where oxic and anoxic prokaryotes might occur in close proximity, and 4) the lack of consideration of confidence intervals on the GOE.

Concerning calibration methodology, our novel consensus approach based on the molecular record (Supplementary Tables S1–S3) addresses many of the known biases associated with calibrating early eukaryotic divergences. Excessive minimum calibrations with narrow uncertainty densities and the application of poorly justified and overly-young maximum calibrations may have adversely affected some of the earlier estimates (Warnock et al., 2012; Battistuzzi et al., 2015). For example, one recent study (Betts et al., 2018) that proposed an origin of modern eukaryotes as late as 1.21 Ga had established a minimum and maximum age for metazoans of 550 and 833 mya, respectively. However, this calibration fell into doubt 3 years later with the discovery of a possible 890 million-year-old sponge (metazoan) fossil (Turner, 2021). One subsequent author considers it to be a trace fossil of a metazoan rather than a whole body fossil (McMenamin, 2023), but this would not undermine its value as a minimum calibration for Metazoa here. Other authors (McMenamin and Kris, 2021; Neuweiler et al., 2022) do not rule out that this fossil may be a keratose sponge, but propose verification for that and all other keratose sponge fossils. We propose that maximum calibrations tied to the fossil record are rarely advisable, as new discoveries may quickly render them questionable.

Recent studies (Betts et al., 2018; Mills et al., 2022) have given LECA significance as the first “modern” eukaryote, neglecting its phylogenetic position at the end of the stem branch of eukaryotes, the point at which eukaryogenesis must have already concluded. This artificially creates a disconnect between the GOE and the origin of eukaryotes. Authors have reported an average time for LECA of 1.55 (2.13–1.09) Ga (Hedges et al., 2004; Parfrey et al., 2011; Betts et al., 2018; Strassert et al., 2021; Wang and Luo, 2021), with several reporting times younger than 1.20 Ga (Douzery et al., 2004; Berney and Pawlowski, 2006; Chernikova et al., 2011). Attempting to characterize eukaryogenesis by presenting point estimates of LECA, as opposed to inferring an interval between FECA and LECA, automatically biases eukaryogenesis towards the recent. LECA is by definition a crown group node and its biological significance in terms of oxygen dependence, or other attributes, compared with extinct eukaryote lineages preceding LECA, is unknown.

Confidence intervals have been used routinely with molecular clock dates for decades, but the same has not been true for the dating of geological events such as the GOE. The proxies used to temporally delineate the bounds of the GOE have been treated largely as data points without statistical confidence intervals. The recent application of confidence intervals on the GOE (Hodgskiss and Sperling, 2022) has corrected this deficiency, expanding the possible time interval for the event considerably (Figure 2C).

With regards to the environment of eukaryogenesis, a spatial disconnect has been proposed for eukaryogenesis and oxygen (Mills et al., 2022). These authors claim that because eukaryotes arose from an Asgard archaeal host (Lazcano and Peretó, 2021), and modern Asgard group Archaea were initially identified from deep oceanic hydrothermal vents, far removed from accessible atmospheric oxygen (Woese and Fox, 1977; Jørgensen et al., 2013), then oxygen may not have been a prerequisite for eukaryogenesis. However, the existence of modern hydrothermal vents located near and at the surface, such as those in Iceland today (Hannington et al., 2001), indicates that habitats suitable for Asgard Archaea need not have been in the deep ocean exclusively. Furthermore, Asgard Archaea are being found in an increasing diversity of environments (Da Cunha et al., 2022) weakening the link with anoxic environments. Therefore, eukaryogenesis may have occurred in or near an oxygenated environment, undermining the proposed spatial disconnect.

Separate from the question of the location and timing of eukaryogenesis is whether the diversification of eukaryotes followed a similar pattern to the two-step rise in atmospheric oxygen. Our reconstruction for the rise in global biological complexity (Figure 3) shows an initial sharp increase followed in the late Proterozoic by a second major increase close to the time of the second increase in oxygen. The result is a concave profile of complexity, rather than one that shows a straight, linear increase. The same two-step pattern has been found in maximum body size, supporting the theory that changes in the atmosphere may have been an influence (Payne et al., 2009). While this trend is compelling, it is important to consider that both data sets (biological and geological) currently have limitations. Complexity data are missing from many taxa, and the precise rise in oxygen through time is still an active area of research (Mänd et al., 2020). Nevertheless, this similarity in the pattern of increase in complexity and oxygen availability seems to suggest that the two are linked.

We have shown evidence to support the theory that the timing of eukaryogenesis was temporally proximate to the oxygenation of our biosphere. This is consistent with the theory that the rise in the global complexity of life was influenced by the rise in oxygen, which would have provided a rich source of cellular energy (Bonner, 2009). While inferences of such ancient events are inherently prone to uncertainty, our new approach offers a potentially useful insight into one of the defining events in the history of Earth’s biodiversity.

## Methodological details

### Calibrations

In all phylogenetic analyses, we used consensus calibrations from the molecular record, as opposed to the fossil record, derived from the 4,000+ studies comprising *TimeTree* (Kumar et al., 2022). For each node of interest (Table 1, Supplementary Tables S1, S2 for the tree of bacterial-origin proteins; Table 2, Supplementary Table S3 for the archaeal tree), we performed a divergence time search in *TimeTree*, generating a sample of published times. We removed any times that were published prior to the year 2000, before which phylogenetic methods and datasets were substantially less developed than they are at present, did not include relaxed clock methods, or were redundant with other studies publishing the same tree. We further removed any times that conflicted with the recent discovery (Turner, 2021) of a possible 890 million-year-old fossil sponge (Peterson et al., 2004; Peterson et al., 2008; Berney and Pawlowski, 2006; Cartwright and Collins, 2007; Berbee and Taylor, 2010; dos Reis et al., 2015; Gold et al., 2015; Schwentner and Bosch, 2015). This fossil has not been credibly rejected by the field, and its phylogenetic position with respect to our nodes of interest make it highly influential to inferring the eukaryogenesis interval. Thus, excluding estimates of this time made prior to its discovery is necessary to reflect the fossil record accurately in this case. Based on the refined sample of published times at each node, we constructed a consensus calibration in the form of a uniform distribution defined by the upper and lower 99% confidence interval around the mean published time, avoiding any biases associated with the shape of the distribution or the application of soft boundaries.

We tested two calibration schemes for the tree of bacterial-origin proteins, one constraining nine node times throughout the tree, and a minimal scheme constraining only the root (LUCA) and the two nodes bracketing the stem time of eukaryotes, which represents the closest divergence to the eukaryogenesis event among extant taxa (Table 1). We found minimal difference between the timing of the eukaryogenesis nodes inferred by each approach and thus we used times from the three-node scheme to infer the timing of eukaryogenesis (Supplementary Table S4).

In the case of the phylogeny of archaeal-origin proteins, we analyzed a published alignment of 28 core proteins (Betts et al., 2018), and thus took the topology inferred in that study as a prior. Given our focus exclusively on the node of eukaryogenesis, we used a minimal calibration scheme as above, including only LUCA and the two nodes bracketing the stem time of eukaryotes, in this case the stem age of Alphaproteobacteria (defined as their divergence from their closest relative in this topology, the large clade containing Campylobacter) and the most well-studied, topologically uncontroversial early divergence within eukaryotes, the crown of Metazoa (Table 2). As with the bacterial-proteins tree, this allowed us to infer the timing of eukaryogenesis as accurately as possible while avoiding biases arising from topological uncertainty and calibrations elsewhere in the tree.

### Phylogeny of bacterial-origin proteins

Our approach aimed to avoid the problems encountered in previous bacterial-gene phylogenies of early eukaryotic species (Derelle and Lang, 2012; He et al., 2014) by attempting to resolve only the nodes necessary to infer the timing of eukaryogenesis, with as few extraneous taxa as possible to mitigate error stemming from taxonomic uncertainty. We first selected a set of 22 complete reference proteomes from NCBI RefSeq (O’Leary et al., 2016). These taxa were selected to provide high resolution for the eukaryogenesis node while minimizing the effects of topological and chronological uncertainty among deep bacterial divergences. We selected three Terrabacteria as outgroups (one *Actinomyces* plus representatives of the two type genera of the Deinococcus-Thermus clade), and two representatives each of the Alpha-, Beta-, and Gammaproteobacteria, providing high taxonomic resolution within the closest bacterial relatives of eukaryotes. We then select*e*d thirteen eukaryotes representing three Viridiplantae (*Arabidopsis thaliana,* the moss *Physcomitrella patens,* and the green algae *Chloropicon*), five animals (human, chicken, zebrafish, fruitfly and the sponge *Amphimedon*) as well as several eukaryotes including *Giardia*, *Leishmania*, *Andalucia*, and the red alga *Porphyra* whose crown divergences are inferred to be more phylogenetically proximate to the true FECA node. Thus, our OTUs were chosen to minimize the risk of taxonomic uncertainty and maximize our ability to time the eukaryogenesis event.

We then ran ProteinOrtho (Lechner et al., 2011) through the Galaxy web platform (Jalili et al., 2020) on this set of proteomes to detect orthologous protein families. We filtered this set to only retain those for which 80% or more of the original species were present. Next, we discarded any proteins associated with the production and maintenance of RNAs and DNA, as these are commonly inferred to be archaeal in origin. This left us with 51 high-coverage protein orthogroups of inferred bacterial-origin. We then removed any redundant isoforms or other duplicates such that each species present was represented by only a single copy of the protein. We aligned these by MUSCLE (Edgar, 2004) through the Galaxy web platform and generated maximum likelihood phylogenies for each using Fastree (Price et al., 2010), which were used to identify any proteins which did not recover monophyletic eukaryotes, indicative of a complex evolutionary history not suitable for use in a concatenated alignment. We then aligned and concatenated the remaining 30 orthogroups. We used MrBayes (Ronquist et al., 2012) to jointly infer the topology and timing of these 22 species under a Thorne-Kishino model (*tk02*) (Thorne and Kishino, 2002) with a lognormal clock rate parameter for a total of one million generations.

### Phylogeny of archaeal-origin proteins

Consistent with other published efforts, we also built a phylogeny to time eukaryogenesis using proteins of inferred archaeal origin. We used a published alignment of 28 core proteins (Betts et al., 2018) for this phylogeny, then ran GBLOCKs (Castresana, 2000) using relaxed settings, given that the defaults are better tuned to identify conservation across more recent divergences. We set a minimum number of conserved and flanking positions set to 52, maximum contiguous nonconserved positions of 32,000, and a minimum block length of 2. We set the allowed gap positions to “with half” and used similarity matrices. These settings allowed us to reduce the published alignment of 20,415 sites (Betts et al., 2018) to 7,990 sites, the latter being more conserved, while also deleting instances of substantial missing data and incorrect alignment. Removing such uninformative or misleading sites has been shown to improve phylogenetic signal and the phylogeny inferred from such an alignment (Talavera and Castresana, 2007). We then inferred a phylogeny with Mr Bayes using the same settings as above with new consensus calibrations (Table 2).

### The eukaryogenesis interval

Because the two closest prokaryotic relatives of eukaryotes are almost certainly extinct, it is not possible to time eukaryogenesis at a single node on any timetree. Instead, we defined the consensus interval within which eukaryogenesis occurred. The older boundary of this interval is established by the divergences of eukaryotes from their closest living archaeal (LACA-K) and bacterial (LBCA-K) relatives, while the younger boundary is established by the oldest unambiguous evidence of the existence of eukaryotes, either in the form of total group eukaryotic fossils or the first divergence among modern eukaryotes (the crown group, LECA). Because phylogenies based on proteins of archaeal and bacterial origin yield different topologies, the number of possible nodes used to estimate the older boundary of the eukaryogenesis interval is large (Figure 2C). In order to estimate the interval of eukaryogenesis as precisely as possible, we identify the youngest possible node to establish the older boundary, and the oldest possible point (either fossil or phylogenetic node) to define the younger boundary. In the case of the older boundary, we report the older 95% HPD, and in the case of the younger boundary, we report the younger 95% HPD to avoid false precision. This approach yields the most precise interval of eukaryogenesis possible, based on molecular clocks and the fossil record.

Importantly, the stem divergence time of Asgard Archaea has been inferred to be older than that of stem Alphaproteobacteria. This makes intervals bracketed by LACA-K wider than those bracketed by LBCA-K. In a phylogeny of three domains, it is possible to time both the stem divergence of Asgard Archaea and that of Alphaproteobacteria in addition to either LACA-K (if the tree is built from archaeal genes) or LBCA-K (if the tree is built from bacterial genes). Thus, in phylogenies built from genes of archaeal origin, eukaryotes will be recovered as sisters to archaea, making it possible to time LACA-K but not LBCA-K, despite this time likely being older than the stem divergence time of Alphaproteobacteria. In these cases, the interval of eukaryogenesis can be bracketed by the alphaproteobacterial stem, disregarding the LACA-K divergence, despite the topological disjunct (Figure 2).

### Complexity metric

The pattern of increase and even how to measure complexity have long been debated (Bonner, 1988; McShea, 1991; Maynard Smith and Szathmáry, 1995). Three universally-applicable metrics of complexity show promise: the number of cell types, genes, and nucleotide base pairs in the haploid genome. The number of unique cell types has long been the primary measure of complexity (Bonner, 1988), ranging from only one in unicellular prokaryotes to over 100 in humans (Bonner, 1988; Valentine et al., 1994; Bell and Mooers, 1997; Carroll, 2001; Newman, 2020). Although precise counts have been a challenge in the past, new methods have been developed to further increase the usefulness of this metric (Márquez-Zacarías et al., 2021). We assembled these data for all families based on Bonner (1988)’s data, and those from two other published accounts (Valentine, Collins and Meyer, 1994; Bell and Mooers, 1997). Because the taxonomic categories used in each of these studies were broad, we were able to estimate the number of cell types for all families in our phylogeny.

Reliable counts of the number of genes and base pairs (genome size) across the tree of life were largely unavailable until the last two decades. As metrics of complexity, neither is ideal because of the confounding factors of whole genome duplication, the proliferation of non-coding DNA, and alternative splicing (Choi et al., 2020). Nonetheless, gene number and genome size are universal and widely available data, and are comprehensive because they represent the entire genetic complement of an organism. Because most of the genome of eukaryotes is non-coding DNA and there is evidence that some or most non-coding DNA has a function (Ariel and Manavella, 2021; Deogharia and Gurha, 2022), gene number and genome size represent distinctly different metrics.

Protein count data were obtained from the JGI GOLD database (Mukherjee et al., 2021) with the following search criteria for all three domains: analysis project, study, and organism type all set to “public,” organism type set to “natural,” study type set to “genome analysis (isolate).” For the bacteria, for which data were far more abundant, we additionally filtered for published studies with organisms that possessed a GenBank ID. We then used these results to score the average number of proteins for as many families as possible in our phylogeny. Genome size data were obtained from the NCBI Genome database (Rédei and dos Reis, 2008). We then used these results to calculate the average genome size in Mb for as many families as possible in our phylogeny.

As a backbone phylogeny on which to reconstruct metrics of complexity, we used *TimeTree*, a global-scale phylogeny available freely online. The *TimeTree* phylogeny was assembled from 4,075 published studies and included 137,306 species. We accessed a beta build of the family-level phylogeny with 5,825 families, which differs minimally from the published fifth edition (Kumar et al., 2022). All metrics of complexity were scored for as many of the tips of this tree as the complexity datasets allowed. We then used PhyloPars in R (Goolsby et al., 2017; Core Development Team, 2020) to impute missing data and perform a maximum likelihood ancestral state reconstruction for each metric of complexity. We then normalized the ancestral state estimates for each taxon by the mean, took the logarithm of each, and averaged them to generate a metric of complexity. We then plotted mean complexity at every node against the age of that node to assess the pattern of complexity through time. To calculate the maximum value of complexity for each time, we constructed a smoothed moving-maximum curve with a set of overlapping 500-My time bins, offset by 100 million years each. Then, the maximum complexity of any node found within each bin at the time defined by the midpoint of that bin was reported, minus a 100-My offset.

## Data Availability

The original contributions presented in the study are included in the article/Supplementary Material, further inquiries can be directed to the corresponding author.
